# Neutrophil expression of glucocorticoid-induced leucine zipper (GILZ) anti-inflammatory protein is associated with acute respiratory distress syndrome severity

**DOI:** 10.1186/s13613-016-0210-0

**Published:** 2016-11-02

**Authors:** Marie-Alix Espinasse, David Hajage, Philippe Montravers, Pascale Piednoir, Guillaume Dufour, Florence Tubach, Vanessa Granger, Luc de Chaisemartin, Benoît Noël, Marc Pallardy, Sylvie Chollet-Martin, Armelle Biola-Vidamment

**Affiliations:** 1 INSERM UMR-996 - Inflammation, Chemokines and Immunopathology, Univ Paris-Sud, Faculté de pharmacie, Université Paris-Saclay, 5 rue JB Clément, 92296 Châtenay-Malabry Cedex, France; 2 Département d’Anesthésie-Réanimation, Hôpital Bichat-Claude Bernard, AP-HP, Paris, France; 3 Université Paris Diderot, Sorbonne Paris Cité, Paris, France; 4 Département d’Epidémiologie et Recherche Clinique, Assistance Publique-Hôpitaux de Paris Hôpital Bichat, INSERM, CIE 801, Université Paris Diderot, Sorbonne Paris Cité, Paris, France; 5 Laboratoire d’immunologie, «Autoimmunité et hypersensibilités», Hôpital Bichat-Claude Bernard, AP-HP, Paris, France

**Keywords:** Neutrophils, Glucocorticoid-induced leucine zipper (GILZ), ARDS, Extracellular DNA, Annexin A1

## Abstract

**Background:**

Glucocorticoid-induced leucine zipper (GILZ) is a potent anti-inflammatory protein involved in neutrophil apoptosis and the resolution of inflammation. Given the numerous pathophysiologic roles of neutrophils in the acute respiratory distress syndrome (ARDS), we postulated that neutrophil GILZ expression might be induced during ARDS, to modulate the inflammatory process and participate in lung repair.

**Methods:**

This single-center, prospective, observational cohort study took place in the surgical intensive care unit of Bichat Hospital (Paris, France) and involved 17 ARDS patients meeting the Berlin criteria at inclusion, and 14 ventilated controls without ARDS. Serial blood samples were obtained every 2 days until extubation or death (from 1 to 9 samples per patient). GILZ protein and gene expression was quantified in blood neutrophils, along with markers of inflammation (CRP, extracellular DNA) or its resolution (Annexin A1).

**Results:**

Neutrophil GILZ expression was detected at the transcriptional and/or translational level in 9/17 ARDS patients (in particular 7/10 severe ARDS) and in 2/14 ventilated controls. The highest mRNA levels were observed in the most severely ill patients (*p* < 0.028). GILZ was expressed in about ¾ of the corticosteroid-treated patients and its expression could also occur independently of corticosteroids, suggesting that inflammatory signals may also induce neutrophil GILZ expression in vivo.

**Conclusions:**

In this pilot study, we show for the first time that blood neutrophils from patients with ARDS can express GILZ, in keeping with an anti-inflammatory and regulatory endogenous role of GILZ in humans. Contrary to some markers of inflammation or its resolution, the levels of *gilz* gene expression were related to ARDS severity.

## Background

The acute respiratory distress syndrome (ARDS) is one of the most severe forms of acute respiratory failure [1] characterized by diffuse alveolar damage. High alveolar concentrations of inflammatory mediators trigger excessive neutrophil influx and activation, leading to the release of reactive oxygen species (ROS), proteases, cytokines and neutrophil extracellular traps (NETs), all of which can contribute to alveolar injury [2]. Both circulating and alveolar neutrophils exhibit delayed apoptosis in this setting, and this is associated with lung injury, impaired resolution of inflammation, clinical severity, and poor outcome [3, 4].

Despite significant research, ARDS management still consists mainly of supportive care, based on optimized ventilation and extracorporeal membrane oxygenation (ECMO) [5]. New therapeutic strategies based on modulation of inflammation, using mesenchymal stromal cells, miRNA, or regulatory T cells, have recently been proposed [6–8]. Steroids have controversial effects when used early during the course of ARDS [9], but have been shown to reduce fibroblast proliferation and cytokine release when used in the late phase, thereby improving recovery [10]. Another effect of steroid therapy might be the induction of glucocorticoid-induced leucine zipper (GILZ).

GILZ is an immunomodulatory protein belonging to the transforming growth factor-β-stimulating clone 22 domain (TSC-22D) family [11]. GILZ can also be induced in vitro by TGF-β and IL-10 [12], and has been detected both in a murine model of arthritis and in the joints of patients with rheumatoid arthritis [13]. These recent in vivo findings suggest that, in some inflammatory conditions, GILZ may be induced independent from exogenous corticosteroid administration.

In vitro, GILZ regulates major inflammatory signal transduction pathways such as activator protein 1 (AP-1), nuclear factor-kappa B (NF-кB), and forkhead box O (FOXO) [11, 14]. GILZ has also been implicated in the control of apoptosis and cell survival in various cell types [11, 15, 16]. We recently showed that GILZ expression by human neutrophils in vitro promotes their apoptosis [17]. These results were confirmed in vivo by Vago et al. [18] who found that preventive or therapeutic administration of the cell-permeable TAT-GILZ fusion protein improved resolution in a mouse model of LPS-induced pleurisy. GILZ thus appears to be an important regulator of neutrophil apoptosis in vitro and in vivo, contributing to the resolution of inflammation. The mechanisms of GILZ action involve the well-described anti-inflammatory and pro-resolving factor Annexin A1 [18, 19]. Indeed, endogenous Annexin A1 induces neutrophils to undergo apoptosis and promotes their efferocytosis by macrophages, as demonstrated in vivo in the mouse model of LPS-induced pleurisy [20].

We postulated that neutrophil GILZ expression might be induced during ARDS, to modulate the inflammatory process and participate in lung repair. The aim of this pilot study was thus to analyze the kinetics of blood neutrophil GILZ expression at the protein and mRNA levels in ventilated septic patients with and without ARDS, and treated or not by corticosteroids. The levels of *gilz* mRNA were then analyzed according to ARDS severity, in comparison with some markers of inflammation (circulating CRP and extracellular DNA) or its resolution (Annexin A1), to assess whether GILZ might be included in a new biological signature of ARDS severity.

## Methods

### Study setting and population

We conducted a single-center, prospective, pilot, observational cohort study of ARDS patients hospitalized in the intensive care unit (ICU) of Bichat Hospital (Paris, France) from January 2012 to March 2013. Patients with HIV infection, hepatitis, transplantation, end-stage cancer, age below 18 years, or ongoing pregnancy were excluded. The protocol was approved by the ethics committee of Paris-Bichat Hospital (CEERB, comité d’évaluation de l’éthique des projets de recherche biomédicale, No. 12-028, Paris, France), and informed consent was obtained from the patients’ legally authorized relatives. Patients mechanically ventilated with a positive end-expiratory pressure above 5 cm H_2_O were prospectively included and classified into two groups: patients with ARDS (*n* = 17) and ARDS-free ventilated controls (*n* = 14), according to the Berlin definition of ARDS (acute hypoxemia, ratio of partial pressure of arterial oxygen (PaO_2_)/fraction of inspired oxygen (FiO_2_) of 300 mmHg or less, and bilateral infiltrates on chest X-ray) [1]. The control group consisted mainly of postoperative care or trauma patients receiving mechanical ventilation for respiratory failure without ARDS criteria. Blood samples were obtained prospectively during routine care, the first within 24 h after ARDS diagnosis or ICU hospitalization and then every 2 days until extubation or death. The following clinical parameters were collected at inclusion: age, sex, reason for ICU admission, CRP plasma level (Dimension Vista^®^1500, Siemens Healthcare diagnostics*, n* < 6 mg/L), ARDS etiology, Simplified Acute Physiology Score II (SAPS II) [21], Sequential Organ Failure Assessment Score (SOFA) [22], Lung Injury Score (LIS) [23], ventilation parameters (PaO_2_/FiO_2_ ratio), and use of extracorporeal membrane oxygenation (ECMO) and other treatments (steroids and vasoactive agents) as recommended by the standard of care of patients with ARDS [24]. ECMO was used as rescue therapy if hypoxemia persisted despite optimal mechanical ventilation, neuromuscular blocking agent administration, and nitric oxide inhalation. Intravenous hydrocortisone hemisuccinate (200 mg/day) was administered in case of severe septic shock with an inadequate response to vascular filling and cardiovascular treatment [25]. Vital status was recorded 28 days after inclusion.

### Blood neutrophil isolation

Neutrophils were isolated immediately after blood sampling by sedimentation on a separating medium containing 5% Dextran T500^®^ (Pharmacia, Uppsala, Sweden) in 0.9% saline and centrifugation on a Ficoll gradient (Eurobio, Les Ulis, France) as previously described [26, 27]. Erythrocytes were removed by hypotonic lysis.

### Immunoblotting of neutrophil-derived GILZ protein

Neutrophils were lysed in cold Laemmli buffer (5% Tris pH 6.8 1.25 M, 10% glycerol, 10% SDS, 1 mM PMSF, 1 mM Na_3_VO_4_, 25 mM β-glycerophosphate, 10 µg/ml aprotinin, 10 µg/ml leupeptin, and 10 µg/ml pepstatin), then boiled, and sonicated. The total protein concentration was measured by bicinchoninic acid assay, and equal amounts of denatured protein were loaded on 12% SDS-PAGE gels and transferred to PVDF membranes. The membranes were incubated with a polyclonal rabbit antibody raised against human GILZ that was developed in our laboratory [15], stripped, and reprobed with an antibody against p38 MAP Kinase (Cell signaling, Danvers, USA, ref 9212) as a loading control.

### Real-time PCR analysis of the neutrophil *gilz* and annexin A1 genes

Neutrophils were lysed in RLT buffer (Qiagen, Courtaboeuf, France) and stored after homogenization of the lysate through a QIAshreder column. Total RNA was isolated with the Qiagen Mini Plus Kit according to the manufacturer’s protocol. RNA integrity was evaluated by capillary electrophoresis using RNA 6000 Nano chips and the Bioanalyzer 2100 (Agilent Technologies). Reverse transcription was carried out as previously described [28]. Real-time PCR analysis was performed using SYBR Green technology on a Biorad CFX96 system with Sso Fast EvaGreen Supermix (Bio-Rad, Marnes la Coquette, France) and appropriate primers for *gilz* [5′-TCTGCTTGGAGGGGATGTGG-3′ and 5′-ACTTGTGGGGATTCGGGAGC-3′] [29], *gapdh* [5′-CAGCCTCAAGATCATCAGC A-3′ and 5′-TGTGGTCATGAGTCCTTCCA-3′], *b2m* (beta-2-microglobulin) [5′-ACCCCCACTGAAAAAGATGA-3′ and 5′-ATCTTCAAACCTCCATGATG-3′], and *annexin A1* (Qiagen QuantiTect primer assay QT00078197), as previously described [28]. The cDNA was amplified with 500-nM final concentrations of each primer, in duplicate 10-µl reactions, by 45 two-step cycles (95 °C 5 s; 60 °C 30 s). “No RT” controls were amplified on all genes to control for genomic DNA contamination, and melting curve analysis was performed to assess the purity of the PCR products. Serial dilutions of a pool of cDNA samples were used to construct linear standard curves, from which PCR efficiencies (E) were calculated for each gene. GeNorm in qBase Plus tool [29] was used to select *b2m* and *gapdh* as reference genes for normalization of mRNA expression results. The normalized relative expression of target genes in samples was determined using the ∆∆*Cq* method with correction for PCR efficiencies, where NRQ = *E*_Target_^−Δ*Cq* Target^ */E*_Ref_^−Δ*Cq* Reference^ and ∆*Cq* = *Cq*_sample_ *−* *Cq*_calibrator_ [30, 31]. Final results were expressed as the *n*-fold differences in target gene expression in a given ARDS patient vs one arbitrarily chosen control patient.

### Circulating extracellular DNA quantification

Circulating DNA was extracted from plasma by using the QiAmp Ultrasens Kit (Qiagen) and stored at −20 °C until use. Cell-free DNA was quantified by fluorimetry in the Quanti-iT™ Picogreen dsDNA assay (Molecular Probes, Eugene, USA) according to the manufacturer’s instructions. The fluorescence signal was recorded on a plate reader (TECAN Infinite^®^ M200, Männedorf, Switzerland).

### Statistical analysis

Demographic data were expressed as the median corrected by the interquartile range (IQR). Fisher’s exact test or the Chi-square test was used to assess the comparability of the study cohort with the control group. Nonparametric variables were compared by using the Mann–Whitney *U* test or the Kruskal–Wallis test for multiple comparisons.

Neutrophil expression of *gilz* and *annexin A1* mRNA and plasma levels of extracellular DNA and CRP were compared between mild/moderate ARDS patients, severe ARDS patients, and controls. A linear mixed-effects regression model was used to take into account the repeated nature of the data and the different times of measurement, and to compensate for data attrition.

In the mixed-effects models, we considered the *gilz* mRNA expression logarithm in order to improve data normality. The 95% confidence interval and corresponding (two-tailed) *p* value were estimated by boostrapping with 1000 replications. A *p* value < 0.05 was considered statistically significant.

We defined an arbitrary positivity threshold for *gilz* mRNA expression as the mean plus twice the standard deviation of the controls’ *gilz* mRNA levels in at least one of the first two serial samples; this value was 2.8 arbitrary units. We used only the first two samples in order to compensate for data attrition, allowing us to analyze an equivalent number of data for each group. Consequently, a “*gilz*-positive” patient had a *gilz* expression level above the threshold in at least one of his/her first two samples. We then analyzed several features of “*gilz*-positive” patients by using the Chi-square test.

All analyses were done using GraphPad software (San Diego, CA, USA) and R version 3.0.2 (R Foundation for Statistical Computing, Vienna, Austria) implemented in the lme4 package version 1.0-4.

The sample size was not calculated in this pilot study because this is the first study to report the *gilz* expression levels in neutrophils from patients with ARDS, and no evidence could be referenced for statistical power analysis. Instead, the sample size was determined by our previous experience.

## Results

### Characteristics of the patients

 A total of 17 ARDS patients and 14 controls were consecutively enrolled. Their main characteristics are shown in Table 1. The ARDS and controls had similar general severity scores (SAPS II and SOFA). The reasons for ICU admissions were similar (mainly sepsis, postoperative care, or trauma). The ARDS patients stayed longer in the ICU than the controls, allowing serial blood sampling for GILZ analysis.

**Table 1:** Characteristics of the patients

Patients	Ventilated controls (*n* = 14)	Mild (*n* = 1) and moderate (*n* = 6) ARDS	Severe ARDS (*n* = 10)	*p* value
Age (years, median ± IQR)	56 ± 19	52 ± 24	59 ± 6	0.82
Male/female (number)	9/5	6/1	7/3	0.78
Comorbidities				
Smoking (number, percentage)	4 (29%)	5 (71%)	2 (20%)	0.07
Obstructive airway diseases (number, percentage)	5 (36%)	5 (71%)	4 (40%)	0.27
Cardiovascular disease (number, percentage)	1 (7%)	1 (14%)	–	0.49
Diabetes (number, percentage)	2 (14%)	1 (14%)	–	0.45
Malignancy (number, percentage)	–	1 (14%)	1 (10%)	0.39
Reasons for ICU admission				
Sepsis (number, percentage)	7 (50%)	6 (86%)	8 (80%)	0.15
Pneumonia (number, percentage)	5 (36%)	5 (71%)	5 (50%)	0.30
Postoperative care (number, percentage)	11 (79%)	7 (100%)	9 (90%)	0.36
Congestive heart failure (number, percentage)	2 (14%)	1 (14%)	–	0.45
Hemorrhagic shock (number, percentage)	1 (14%)	2 (29%)	–	0.13
Trauma (number, percentage)	4 (29%)	1 (14%)	3 (30%)	0.73
SAPS II (median ± IQR)	39.0 ± 13.5	45.0 ± 21.0	43.5 ± 20.5	0.67
SOFA at inclusion (median ± IQR)	6 ± 4	7 ± 2	7.5 ± 3.5	0.06
CRP at inclusion (mg/L, median ± IQR)	217 ± 325	211 ± 195	273 ± 313	0.80
Number of samples per patient (range)	1–5	1–5	1–9	–
Cause of lung injury				
Pneumonia at inclusion (number, percentage)	5 (36%)	5 (71%)	8 (80%)	0.081
Aspiration (number, percentage)	1 (7%)	2 (29%)	3 (30%)	0.29
Septic shock at inclusion (number, percentage)	5 (36%)	6 (86%)	8 (80%)	0.043
Extra pulmonary cause (number, percentage)	–	3 (43%)	2 (20%)	0.038
Steroid therapy^a^ (number, percentage)	1 (7%)	1 (14%)	5 (50%)	0.051
Vasoactive agent therapy^a^ (number, percentage)	7 (50%)	7 (100%)	6 (60%)	0.065
Lung injury score at inclusion (median ± IQR)	0.67 ± 0.91	2.33 ± 0.66	3.00 ± 0.72	<0.0001
Respiratory variables				
Positive end-expiratory pressure, cmH20 (median ± IQR)	5 ± 0.5	8 ± 1	9 ± 2	0.001
PaO_2_/FiO_2_ at inclusion mmHg (median ± IQR)	253 ± 85	210 ± 49	67 ± 29	<0.0001
PaCO_2_ at inclusion mmHg (median ± IQR)	41 ± 5	43 ± 4	43 ± 4	0.72
ECMO^a^ (number, percentage)	–	–	6 (60%)	0.0004
Mortality at day 28 (number, percentage)	4 (28%)	2 (29%)	6 (60%)	0.26

*ARDS* acute respiratory distress syndrome, *LIS* Lung Injury Score [23], *SOFA* Sequential Organ Failure Assessment, *SAPS II* Simplified Acute Physiology Score II [21], *ECMO* Extracorporeal membrane oxygenation, *CRP* C-reactive protein, *IQR* interquartile range

*p* values: determined by Kruskal–Wallis test or Fisher’s exact test for continuous and categorical variables, respectively

^a^During the ICU stay

### The *gilz* gene is transiently expressed by blood neutrophils from ARDS patients, which depends on corticosteroids but not on vasoactive agent administration

**Fig. 1:**
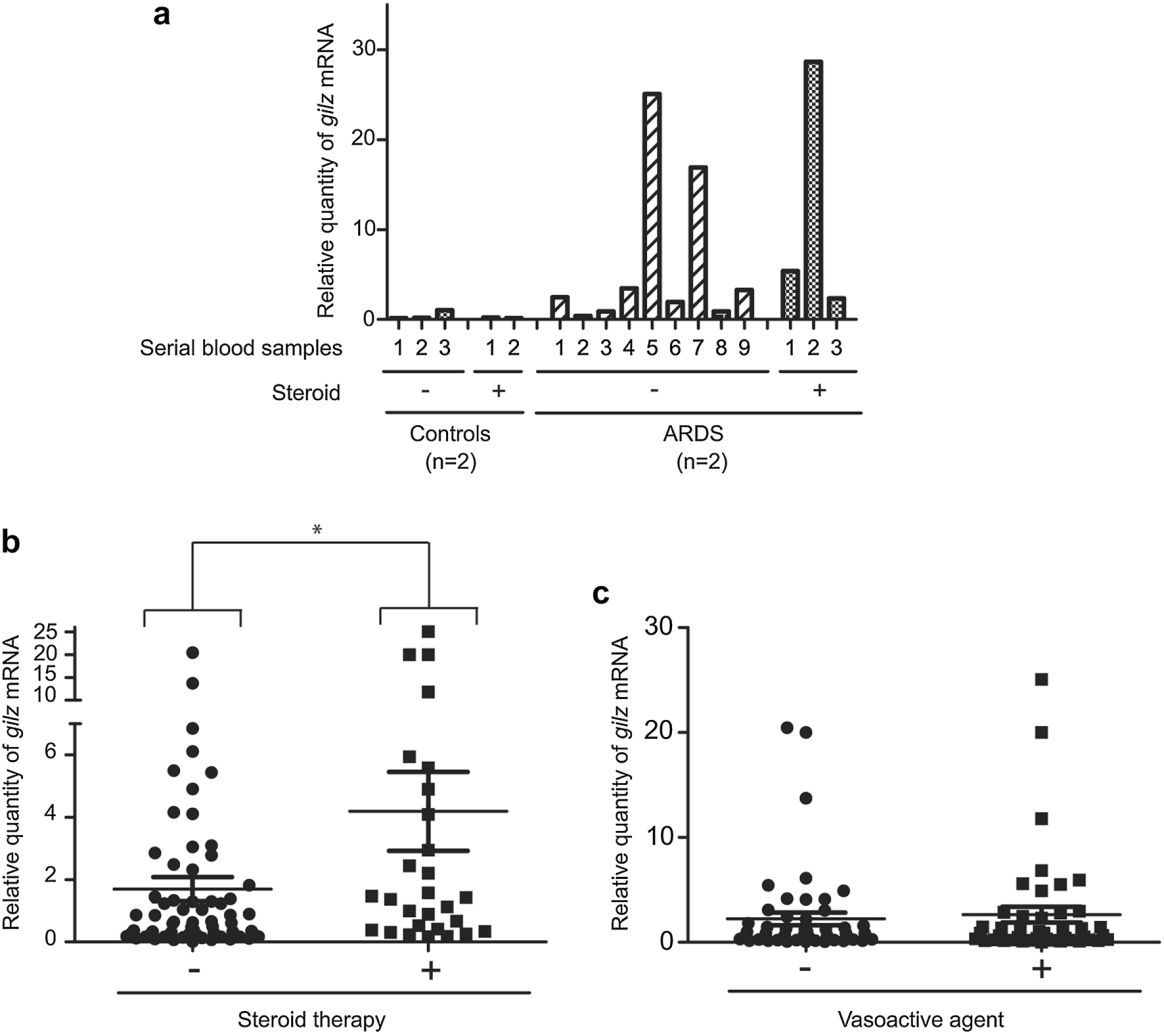
**a** Kinetics of *gilz* mRNA expression in neutrophils from 2 representative controls and 2 representative ARDS patients. The number of serial blood samples obtained (every 2 days until extubation or death) is indicated (*n* = 2–9 per patient, depending on the patient). Some patients received hydrocortisone hemisuccinate (200 mg/day; steroid). Relative quantity of *gilz* mRNA was expressed as *gilz* expression level divided by the mean of *gapdh* and *b2m* levels. **b** Implication of corticoid therapy in *gilz* mRNA expression in all samples studied. Results are expressed as the relative quantity of *gilz* mRNA (mean ± standard error of the mean). *p < 0.05, Mann–Whitney test. **c** Implication of vasoactive agent administration in all samples studied. Results are expressed as the relative quantity of *gilz* mRNA (mean ± standard error of the mean). *ARDS* acute respiratory distress syndrome, *GILZ* glucocorticoid-induced leucine zipper

Neutrophil *gilz* expression was transiently detected in several ARDS patients (9/17) but only in few controls (2/14). Figure 1a shows the time course of *gilz* mRNA expression in two representative controls and two representative ARDS patients. Depending on the patients, 1–9 mRNA samples could be obtained during the ICU stay and analyzed. Neutrophil *gilz* mRNA relative quantity was significantly increased in patients with steroid therapy as compared with patients without corticosteroids (*p* < 0.05) (4.2 ± 6.7 and 1.7 ± 3.2 arbitrary units) (Fig. 1b), but was also evidenced in the absence of corticotherapy (Fig. 1a, b). Vasoactive agents were administered to 65% of the 31 patients*; gilz* expression was similar in patients who did and did not receive vasoactive agents (2.2 ± 4.3 and 2.6 ± 5.0 arbitrary units) (Fig. 1c).

### GILZ protein is transiently expressed by blood neutrophils from ARDS patients

**Fig. 2:**
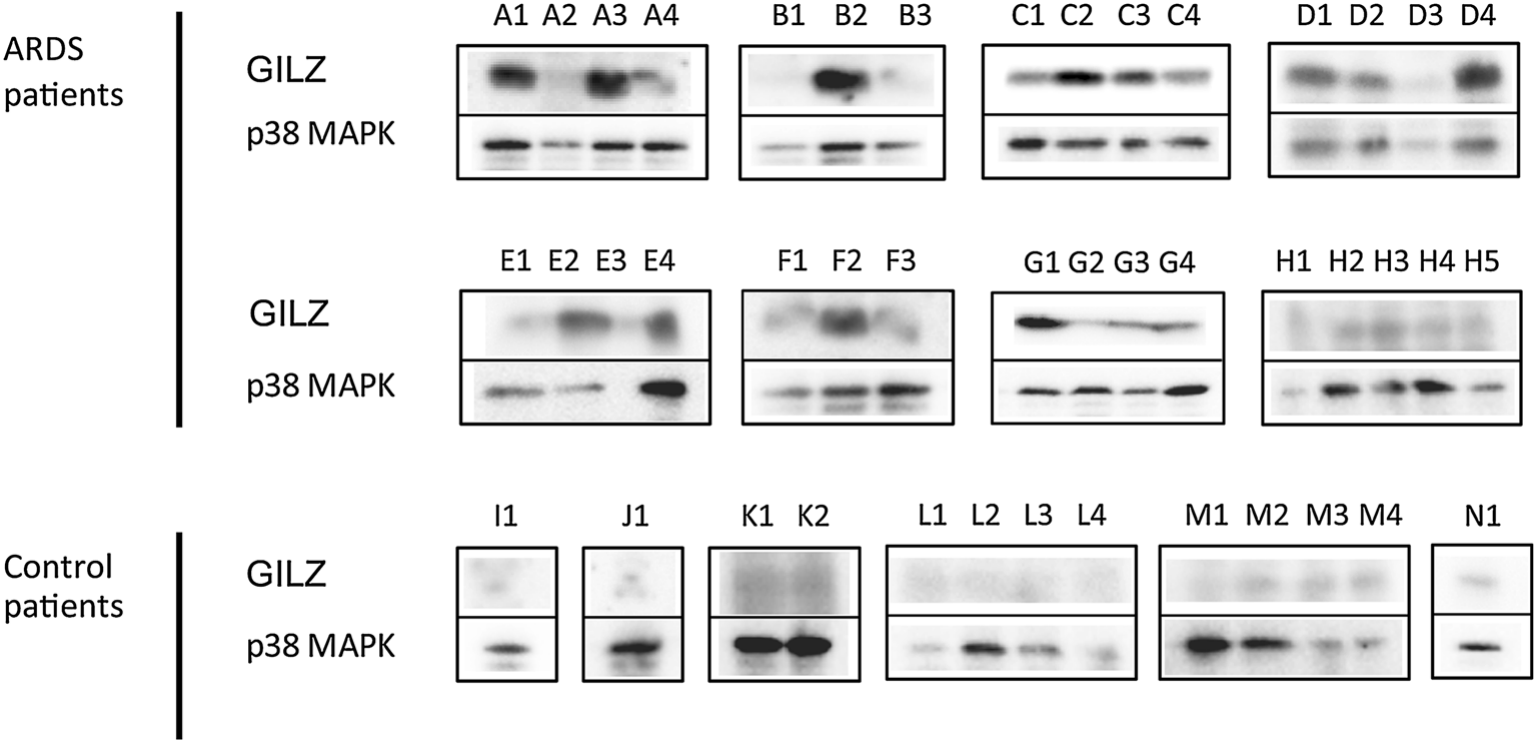
Kinetics of GILZ protein expression in ARDS patients and controls. Representative serial Western blot data for 8 ARDS patients (A to H) and 6 controls (I to N) performed every 2 days. p38 MAPK expression served as control. *A1* sample 1 of patient A, *A2* sample 2 of patient A

GILZ was detected in neutrophil homogenates from 7 upon the 8 ARDS patients that could be studied (good quality protein samples for 47% of the ARDS patients) and was almost undetectable in the 6 controls studied (Fig. 2).

Depending on the patients, 1–5 protein samples could be obtained during the ICU stay and analyzed. GILZ protein was transiently expressed during ARDS, as described above for *gilz* gene expression (Fig. 2).

### Neutrophil *gilz* mRNA levels are related to ARDS severity

**Fig. 3:**
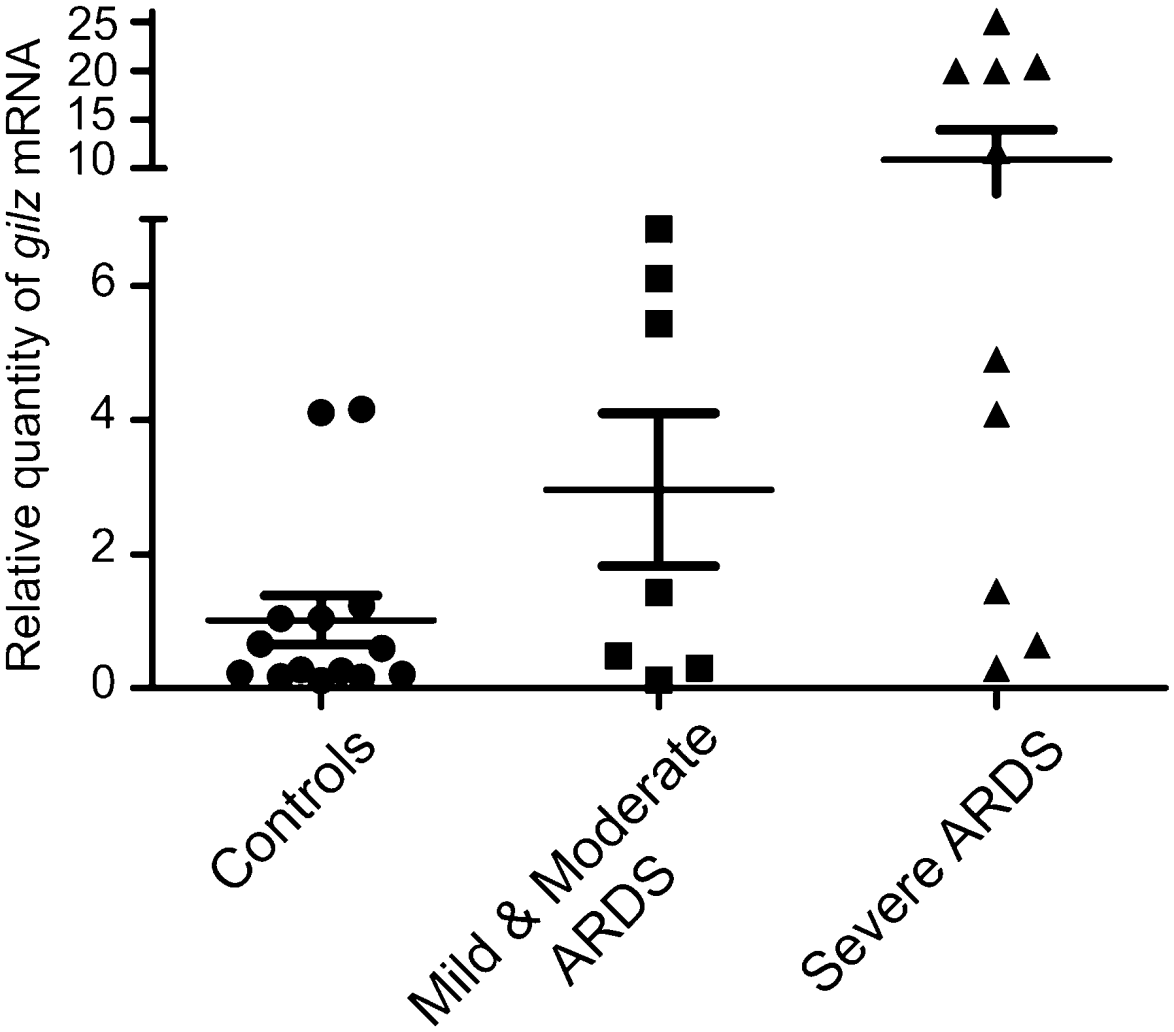
*gilz* mRNA expression in the neutrophils of ARDS and controls (highest point of *gilz* expression for each patient). Results are expressed as the mean ± standard error of the mean. Relative quantity of *gilz* mRNA was expressed as *gilz* expression level divided by the mean of *gapdh* and *b2m* levels. *ARDS* acute respiratory distress syndrome, *GILZ* glucocorticoid-induced leucine zipper

**Fig. 4:**
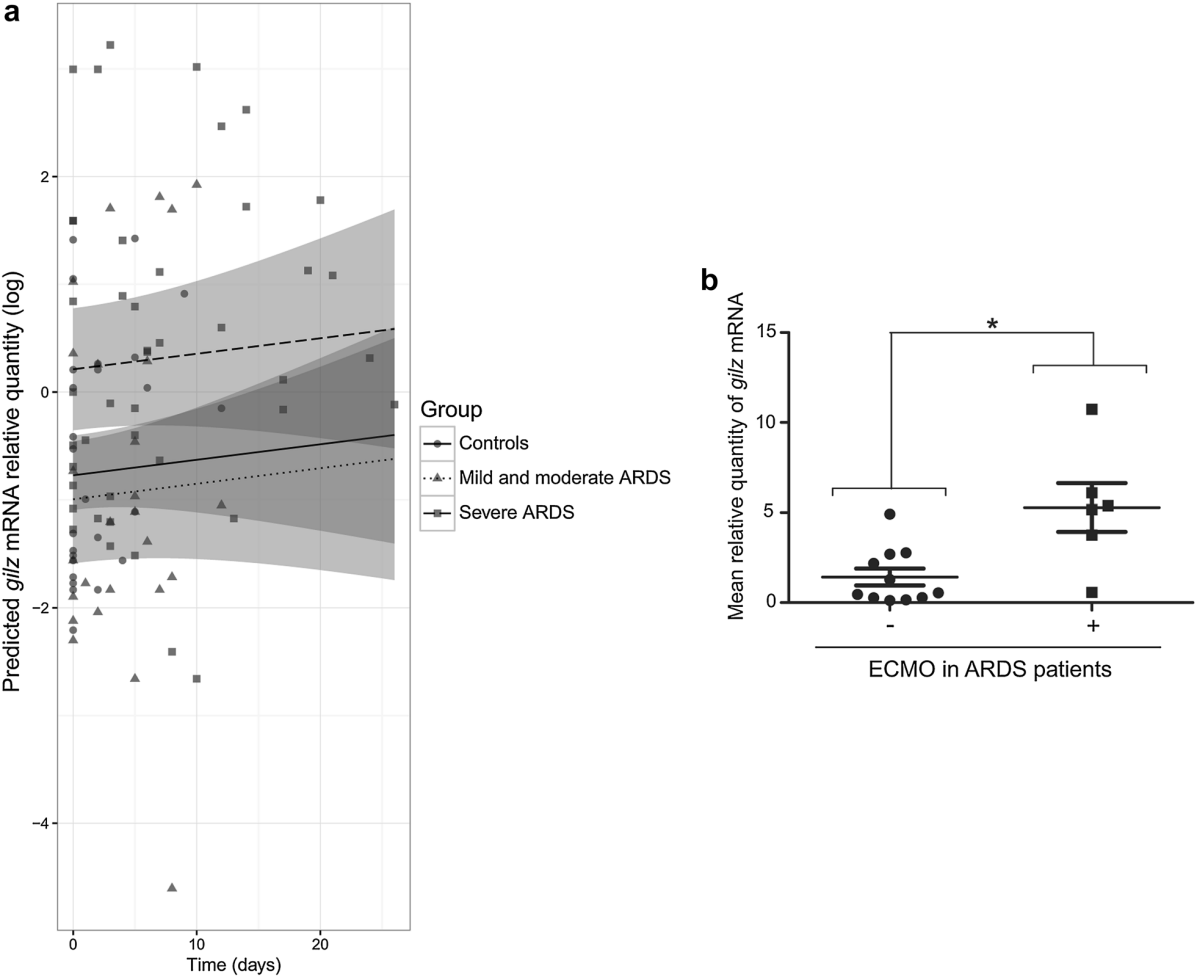
*gilz* gene expression in relation to ARDS severity. **a** *gilz* expression levels predicted by a linear mixed model. Controls (full line), mild/moderate ARDS patients (*small dotted line*), and severe ARDS patients (*large dotted line*) are represented. For each prediction, the confidence interval is represented by the *gray region*. Fixed effects of the model are: − 0.773 − 0.222 × mild/moderate ARDS + 0.984 × severe ARDS + 0.014 × time. All values are represented on the graph: *circles* for controls, *triangles* for mild/moderate ARDS patients, and *squares* for severe ARDS patients. **b** Mean *gilz* mRNA expression in neutrophils from ARDS patients with (*n* = 6) and without extracorporeal membrane oxygenation (*n* = 11). Results are expressed as the mean ± standard error of the mean *gilz* mRNA value. (*p < 0.05, Mann–Whitney test)

We then wanted to compare neutrophil *gilz* mRNA levels in controls, mild/moderate ARDS patients, and severe ARDS patients. We first analyzed the highest value of *gilz* expression for each patient and found that it tended to be higher in ARDS patients than in controls, particularly when only severe ARDS patients were considered (Fig. 3). We then used a linear mixed-effects regression model to refine this comparison and to compensate for data attrition linked to differences in the numbers of serial samples analyzed for each patient (*n* = 1–9). This allowed us to calculate predicted *gilz* mRNA levels based on available measured values. As shown in Fig. 4a, predicted *gilz* mRNA levels did not differ between mild/moderate ARDS patients and controls (*p* = 0.665). However, predicted *gilz* mRNA levels were higher in severe ARDS patients than in controls (*p* = 0.035) and mild/moderate ARDS patients (*p* = 0.028) (Fig. 4a). Although *gilz* mRNA expression appeared variable in time, no linear interaction or association with time was found. We then examined whether *gilz* expression was related to the PaO_2_/FiO_2_ ratio and found that the correlation between *gilz* expression and a PaO_2_/FiO_2_ below 100 was very close to statistical significance (*p* = 0.054) (data not shown). Interestingly, *gilz* expression was significantly higher in ARDS patients receiving ECMO than in other ARDS patients (*p* < 0.005) (Fig. 4b).

**Fig. 5:**
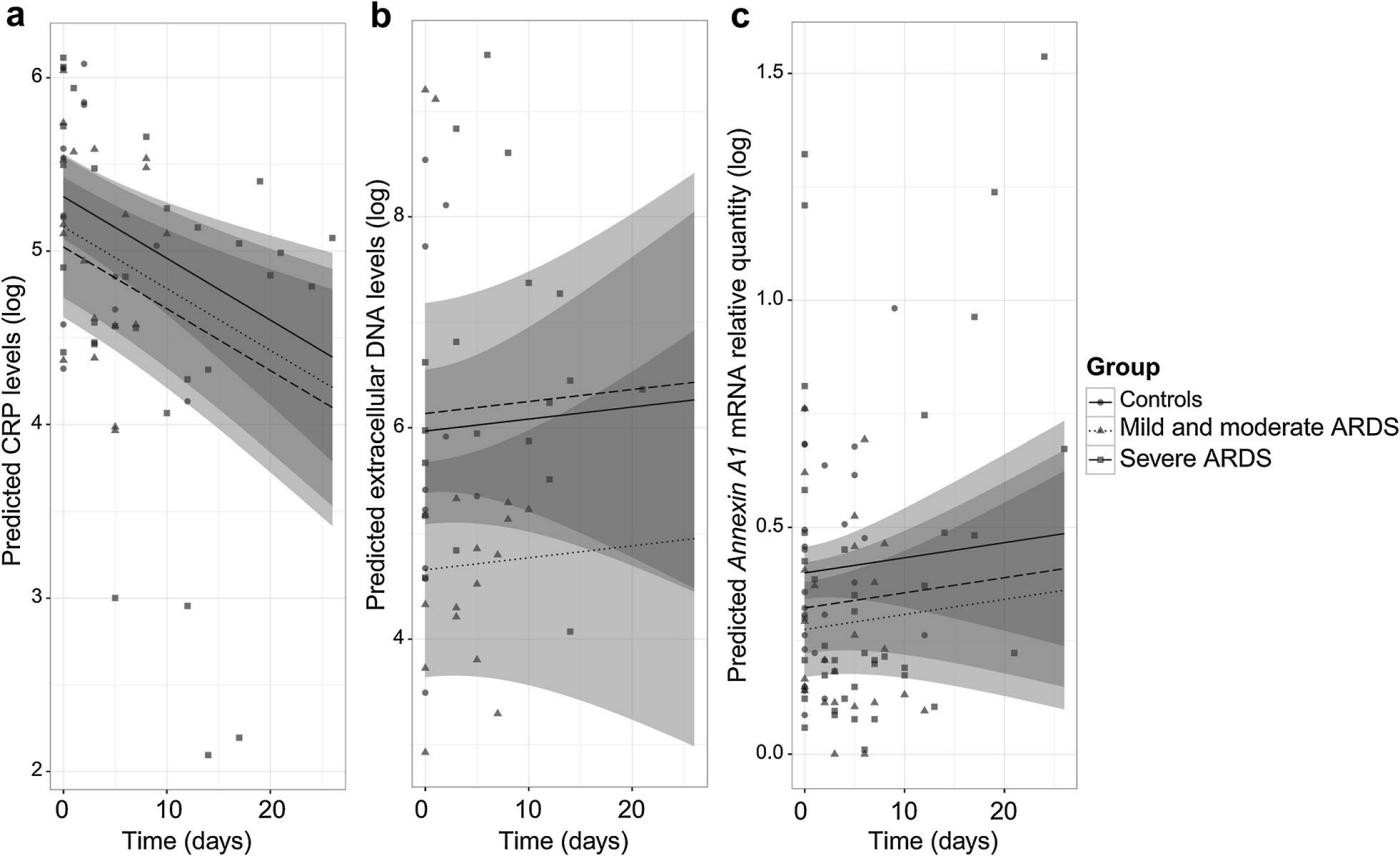
CRP, extracellular DNA and *annexin A1* mRNA levels in relation to ARDS severity. CRP (**a**), extracellular DNA (**b**), and *annexin A1* mRNA (**c**) levels predicted by a linear mixed model. controls (*full line*), mild/moderate ARDS patients (*small dotted line*), and severe ARDS patients (*large dotted line*) are represented. For each prediction, the confidence interval is represented by the *gray region*. All values are represented on the graph: *circles* for controls, *triangles* for mild/moderate ARDS patients, and squares for severe ARDS patients. Fixed effects of the model with the CRP are: 5.314 − 0.175 × mild/moderate ARDS − 0.291 × severe ARDS − 0.036 × time. Fixed effects of the model with DNA are: 5.969 − 1.311 × mild/moderate ARDS + 0.167 × severe ARDS − 0.011 × time. Fixed effects of the model with *annexin A1* are: 0.400 − 0.125 × mild/moderate ARDS − 0.077 × severe ARDS + 0.003 × time

To evaluate GILZ as a possible new marker of ARDS severity, we used the same linear mixed-effects regression model to analyze three other parameters related to inflammation (CRP, extracellular DNA) or its regulation (Annexin A1). We found that predicted circulating CRP levels did not differ across the three patient groups (Fig. 5a) and that *gilz* gene expression did not correlate with CRP levels (data not shown). As extracellular DNA levels have been linked to organ injury in some forms of acute lung injury and sepsis, we measured extracellular DNA in the same samples and found no difference across the three groups of patients (Fig. 5b), confirming that this parameter is not specific for ARDS. Finally, we analyzed neutrophil-derived Annexin A1, as this glucocorticoid-inducible pro-resolving protein can cooperate with GILZ to dampen inflammation. We found that predicted *annexin A1* mRNA levels did not differ across the three groups of patients (Fig. 5c), suggesting that *annexin A1* regulation may occur at a different level than *gilz* regulation.

Together, these results show that GILZ can be expressed by neutrophils during the course of ARDS, at both the protein and gene level, and that its expression level is associated with clinical severity, more specifically than other markers of inflammation or its resolution.

### Clinical definition of “*gilz*-positive” ARDS patients

We then analyzed several features of “*gilz*-positive” patients (see above), including clinical parameters (ARDS, septic shock at inclusion, and pneumonia at inclusion [32]), treatment, and mortality. Using the Chi-square test to assess whether or not two variables were independent, we found that neutrophil *gilz* gene expression was associated with ARDS (*p* < 0.05) but not with septic shock or pneumonia (Table 2). The percentage of “*gilz*-positive” patients was higher in the severe ARDS group (70%) than in the mild/moderate ARDS group (29%) and than in the ventilated controls (14%). Corticosteroid therapy might play a role in *gilz* induction as neutrophils from steroid-treated patients expressed *gilz* in 71% of cases (Table 2). However, these results also suggest that steroid therapy was not required for *gilz* expression and that other inducers could be active. This model also confirmed that neutrophil *gilz* expression was not linked to administration of vasoactive agents (Table 2). Finally, *gilz* positivity was not linked to day-28 mortality among ARDS patients (Table 2).

**Table 2:** Characteristics of “*gilz*-positive” patients

Overall patients *n* = 31	ARDS		Steroids^a^		Vasoactive agents^a^		Pneumonia at inclusion		Septic shock at inclusion		Vital status on day 28	
	No *n* = 14	Yes *n* = 17	No *n* = 24	Yes *n* = 7	No *n* = 10	Yes *n* = 21	No *n* = 13	Yes *n* = 18	No *n* = 12	Yes *n* = 19	Dead *n* = 12	Alive *n* = 19
*gilz*-positive patients	2/14 (14%)	9/17 (53%)	6/24 (25%)	5/7 (71%)	3/10 (30%)	8/21 (38%)	3/13 (23%)	8/18 (44%)	3/12 (25%)	8/19 (42%)	4/12 (33%)	7/19 (37%)
*p* value	0.025		0.024		0.660		0.220		0.332		0.842	

“*gilz*-positive” patients had *gilz* mRNA levels above the threshold in at least one of the first two serial samples

*p* values: determined by the Chi-square test

^a^At least one injection

## Discussion

The main findings of this pilot study are that GILZ, a potent regulator of inflammation, can be induced in ARDS patients’ blood neutrophils, that its expression is transient, and that mRNA levels are related to ARDS severity. Therapeutic GILZ upregulation might thus constitute a potential beneficial approach in ARDS.

During ARDS, lung edema and endothelial/epithelial cell damage promote the recruitment and activation of neutrophils in interstitial and bronchoalveolar spaces. The overwhelming inflammatory response associated with ARDS not only targets the lung but also causes persistent elevation of circulating protease and cytokine levels, and primed or hyperactivated neutrophil numbers, leading to extrapulmonary organ dysfunction [2, 33]. The mechanisms that regulate this acute, severe inflammatory response are poorly understood. Neutrophils themselves might help to dampen this inflammation, by releasing IL-1RA or TGF-β, by adopting an immunosuppressive phenotype (CD16^bright^ CD62-L^dim^) or by engaging in crosstalk with other immune cells [34, 35]. Indeed, we have previously shown that neutrophil-derived extracellular traps (NETs) can downregulate dendritic cell activation in vitro, thereby inducing Th2 T lymphocyte polarization [27]. Moreover, a new neutrophil-related gene signature has been described in patients with early sepsis-induced ARDS, based on two anti-inflammatory genes: *OLFM4* (encoding olfactomedin 4) and *LCN2* (encoding lipocalin 2) [36]. The main objective of the present study was to determine whether GILZ, an anti-inflammatory protein that we recently found to be expressed by human neutrophils in particular stimulatory conditions in vitro [17], is modulated during ARDS.

We show for the first time that *gilz* is expressed by blood neutrophils of most severe ARDS patients but in only a minority of mild/moderate ARDS patients or ARDS-free ventilated controls. Interestingly, GILZ expression during ARDS was transient. The very short half-life of *gilz* transcripts, of around 2 h in murine lymphocytes [16] and macrophages [37], would explain, at least in part, this transient expression. Our study provides new insights into the neutrophil-related events, adding a new gene in the recently described signature [36].

Neutrophil *gilz* mRNA levels were related to ARDS severity, being significantly higher in severe ARDS patients (reaching levels of 7–9 times the threshold value) than in mild/moderate ARDS patients and in ventilated controls. Moreover, patients receiving ECMO had higher *gilz* expression than patients without ECMO. This rescue therapy is used in severe ARDS when conventional mechanical ventilation fails [5, 38]. As ECMO has been shown to have pro-inflammatory effects in vivo [39, 40], it is conceivable that GILZ induction was aimed at compensating for these effects.

In order to better document the clinical relevance of *gilz* expression in this setting, we used the linear mixed-effects regression model to compare circulating levels of two classical markers of inflammation (CRP and extracellular DNA) in the three patient groups. CRP levels did not differ across the three groups and did not correlate with the neutrophil *gilz* expression level, suggesting that GILZ expression is not only induced by inflammatory signals. Extracellular DNA levels are a marker of NET release. NETs are decondensed DNA fibers that bear histones and several proteins from the granular and cytoplasmic compartments, which can induce lung damage [41]. We found that circulating extracellular DNA levels were similarly elevated in the three patient groups, independently of clinical severity. This concurs with the results of Yildiz et al. [42] who found that mechanical ventilation induced netosis in an animal model of sepsis, but that netosis had no major pathogenic effect. However, netosis intensity has been linked to severity in two models of sterile ARDS: acid-aspiration-induced ARDS [43] and transfusion-related acute lung injury [44]. *Gilz* expression might thus be a more specific marker of ARDS severity than other markers such as CRP and NETs.

The stimuli leading to neutrophil *gilz* expression in vivo are probably multiple in ARDS. Even if steroid therapy did not appear to be a prerequisite for *gilz* expression, it was observed in almost ¾ of corticosteroid-treated patients. We found a near-significant negative correlation between *gilz* levels and the PaO_2_/FiO_2_ ratio, suggesting that hypoxia could play a role. Consistent with our results, GILZ expression was recently reported to be induced by hypoxia in vitro, in both murine macrophages [45] and human epithelial cells [46]. The presence of areas of hypoxia being a prominent feature of inflamed tissues, GILZ expression could be induced not only by anti-inflammatory signals such as glucocorticoids (GCs), but also by the inflammatory context as previously hypothesized in arthritis [13].

We recently demonstrated that the induction of neutrophil *gilz* expression promotes apoptosis [17]. Moreover, the combination of GCs and hypoxia has also been recently reported to induce neutrophil apoptosis in vitro [47]. Taken together, we could postulate that the induction of neutrophil *gilz* expression by GCs, hypoxia, and/or other inflammatory signals could contribute to neutrophil apoptosis, necessary for lung repair.

Neutrophil *gilz* expression may thus help to resolve inflammation. There is growing interest in the possible role of two GC-induced proteins (Annexin A1 and GILZ) in the anti-inflammatory effects of GCs in vivo. Annexin A1 is upregulated during natural resolution of acute inflammation and has been shown to have pro-resolving and immunosuppressive properties both in vitro [48, 49] and in vivo at the site of inflammation [18]. It was also recently demonstrated that Annexin A1 can control GILZ expression [19], revealing close cooperation between the two proteins in the resolution of inflammation. Here, we found that blood neutrophil *annexin A1* mRNA levels were not linked to ARDS severity, suggesting that *annexin A1* is rather a general marker of inflammation resolution.

An anti-inflammatory role of endogenous GILZ in humans was first suggested by Beaulieu et al. [13] in rheumatoid arthritis. More recently, Vago et al. [18] demonstrated that GILZ administration accelerated and improved resolution of inflammation in a mouse model of acute lung injury (ALI). Interestingly, in a mouse model of LPS-induced ALI, we have found that GILZ is expressed in alveolar neutrophils and peaks when pulmonary inflammation is maximal (Espinasse et al., unpublished observations). GILZ might thus participate in the regulation of inflammation in vivo.

This prospective observational pilot study has several limitations. First, we had no access to alveolar neutrophils. Second, the cohort size was modest and not sufficiently large for biomarker exploration. Third, we did not perform a power analysis because no reference data are available in this setting. Finally, because blood samples were obtained prospectively during usual care of patients until death or extubation, the number of samples is different for each patient and some patients may have a longer follow-up than others. This data attrition can be viewed as a missing data issue, and mixed effect modeling that we used in this study has been found robust in this setting [50], as they implicitly impute outcome trajectories beyond the time of death or extubation. More complex modeling techniques could have been considered in this situation [51], but they were not applicable because of the relatively small sample size of this pilot study.

## Conclusions

We show for the first time that GILZ, a potent regulator of inflammation and an inducer of apoptosis, can be expressed by blood neutrophils of ARDS patients at both the gene and protein level. GILZ expression was transient and could occur without steroid therapy. Interestingly, the highest neutrophil *gilz* expression levels were observed in patients with severe ARDS, and these levels correlated with the PaO_2_/FiO_2_ ratio; neutrophil GILZ may prove to be a potential biomarker for ARDS severity in a larger study. These data also suggest that blood neutrophil activation during ARDS might not only contribute to lung injury but also participate in endogenous inflammation regulation.

## Abbreviations

ARDS: acute respiratory distress syndrome
ALI: acute lung injury
CRP: C-reactive protein
ECMO: extracorporeal membrane oxygenation
FiO_2_: fraction of inspired oxygen
ICU: intensive care unit
GCs: glucocorticoids
GILZ: glucocorticoid-induced leucine zipper
NETs: neutrophil extracellular traps
PaO_2_: partial pressure of arterial oxygen
PEEP: positive end-expiratory pressure
ROS: reactive oxygen species
SAPS II: Simplified Acute Physiology Score II
SOFA: Sequential Organ Failure Assessment Score
SS: septic shock
